# Eva‐1 homolog A promotes papillary thyroid cancer progression and epithelial‐mesenchymal transition via the Hippo signalling pathway

**DOI:** 10.1111/jcmm.15909

**Published:** 2020-09-23

**Authors:** Bang‐Yi Lin, Jia‐Liang Wen, Chen Zheng, Li‐Zhi Lin, Cheng‐Ze Chen, Jin‐Miao Qu

**Affiliations:** ^1^ Department of Surgical Oncology The First Affiliated Hospital of Wenzhou Medical University Wenzhou China

**Keywords:** epithelial‐mesenchymal transition, *EVA1A*, Hippo, intrinsic apoptosis pathway, papillary thyroid cancer

## Abstract

Recently, the incidence of thyroid cancer is increasing worldwide. Papillary thyroid cancer (PTC) is the most common histological type of thyroid cancer. Whole‐transcriptome sequence analysis was performed to further understand the primary molecular mechanisms of the occurrence and progression of PTC. Results showed that Eva‐1 homolog A (EVA1A) may be a potential gene for the PTC‐associated gene in thyroid cancer. In this work, the role of EVA1A expression in thyroid cancer was investigated. Real‐time PCR was performed to detect the expression level of EVA1A in 43 pairs of PTC and four thyroid cancer cell lines. The Cancer Genome Atlas (TCGA) database was used to evaluate the relationship between the expression level of EVA1A and the pathological feature of PTC. The logistic regression analysis of the TCGA data set indicated that the expression of EVA1A was an independent risk factor for tumour, nde and metastasis (TNM) in PTC. This study shows the down‐regulation of EVA1A inhibited the colony formation, proliferation, migration and invasion of PTC cell lines. In the protein level, knockdown of EVA1A can regulate the expression of N‐cadherin, vimentin, Bcl‐xL, Bax, YAP and TAZ. This study indicated that EVA1A was an oncogene associated with PTC.

## 1 BACKGROUND

Thyroid cancer is one of the most common endocrine malignant tumours.^1^ The incidence rates of thyroid cancer are steadily increasing over the past 10 years.^2^ In 2018, 53 990 new cases and 2060 deaths are expected in the United States.^3^ In China, 90 000 new cases and 6800 deaths are estimated by the National Health and Family Planning Commission of China in 2015.^4^ The histological types of thyroid cancer are classified into follicular thyroid cancer (FTC), anaplastic thyroid cancer, papillary thyroid cancer (PTC), poorly differentiated thyroid cancer and parafollicular C cell‐derived medullary thyroid cancer.^5^ PTC is a well‐differentiated thyroid cancer accounting for about 80% of all thyroid cancers.^6^ Despite the good prognosis of thyroid cancer, up to 25% of patients with PTC has a significant risk for recurrence.^7^

EVA1A is a novel endoplasmic reticulum and lysosome‐associated protein‐coding RNA located on chromosome 2p12.^8^ In several cancers, such as hepatocellular carcinoma and non‐small cell lung cancer, the expression of EVA1A is low in carcinoma tissues, and EVA1A can inhibit tumour cell growth through autophagy and apoptosis.^9^, ^10^ In contrast to its expression in hepatocellular carcinoma and non‐small cell lung cancer, the EVA1A expression in thyroid cancer tissues is significantly higher than that in the normal thyroid tissue according to The Cancer Genome Atlas (TCGA) database (*P* < .0001). This phenomenon is also observed in bladder urothelial carcinoma, colon adenocarcinoma and lymphoid neoplasm diffuse large B‐cell lymphoma. (*P* < .05). However, the relationship between EVA1A and thyroid cancer remains poorly understood.

We have previously performed whole‐transcriptome sequencing and bioinformatics analysis on 19 paired PTC tissues and adjacent normal tissues^11^ and found that EVA1A, a gene encoding a transmembrane protein, is one of the most significantly up‐regulated genes among patients with PTC. Through the gene set rich analysis (GSEA), we have found that the expression of EVA1A expression is correlated to metastasis, proliferation and apoptosis. These phenotypes are related to several pathways described below.

The epithelial‐mesenchymal transition (EMT) is a biological phenomenon that plays an essential role in cancer invasion and metastasis.^12^ The levels of the related proteins N‐cadherin and vimentin are up‐regulated during EMT. This process leads to the decrease in cell‐cell adhesion and an increase in tumour cell metastasis and invasion.^13^

The intrinsic apoptosis pathway is regulated by the Bcl‐2 family located in the mitochondria. The antiapoptosis protein Bcl‐xL and the proapoptosis protein Bax are prominent cancer makers. Sufficient evidence has shown that the up‐regulation of Bcl‐xL and the down‐regulation of Bax can suppress the apoptosis of the tumour cells.^14^

The Hippo signalling pathway plays an essential role in regulating tumour cell proliferation and apoptosis.^15^ As a pathway that suppresses tumours, the Hippo signalling pathway inhibits the transcriptional activity of the Yes‐associated protein (YAP) and the transcriptional coactivator with PDZ‐binding motif (TAZ). YAP and TAZ are downstream effectors of the Hippo pathway and overexpressed in numerous cancers.^16^ In recent years, several studies have found that the Hippo signalling pathway can promote tumour invasion and metastasis via EMT.^16^, ^17^, ^18^, ^19^ And the molecular mechanism through which the Hippo pathway activates apoptosis is realized via p73.^20^, ^21^, ^22^ According to researchers, such as Yoon et al,^23^ the tumour suppressor p73 mediates apoptosis by regulating the antiapoptosis regulator Bcl‐xL. Similar to this idea, Wolf et al^24^ have found that p73 can form a complex with p53, which results in the induction of apoptosis through Bax via the phosphorylation of the Thr81 of p53. These studies indicate that the Hippo pathway regulates apoptosis via Bcl‐xL and Bax.

In this study, the expression of EVA1A in 43 thyroid cancer tissues and paired normal tissues was detected using the quantitative real‐time PCR (qRT‐PCR) to verify the results of transcriptome sequencing. After the silencing of the EVA1A gene by the siRNA in the human PTC cell lines, in vitro cell biology experiments and Western blot analysis were performed. This work aimed to determine the relationship between EVA1A expression and its role in the proliferation, metastasis and apoptosis of thyroid carcinoma.

## 2 MATERIALS AND METHODS

### 2.1 Patients and samples

This study included 43 patients with PTC who underwent surgical treatment at The First Affiliated Hospital of Wenzhou Medical University between May 2018 and April 2019. The final diagnoses of the patients were confirmed by post‐operative pathological examinations. The samples of the paracarcinoma thyroid tissue (approximately 10 mm distance from the tumour) of 44 patients were used as controls. The samples were obtained at the time of initial surgery, frozen in liquid nitrogen, and stored at −80°C. Besides, none of the patients was suffering from pre‐operative managements, such as chemotherapy or radiotherapy. Patients agreed on the use of human tissues and signed the informed consent and study protocol, which was provided by the Ethics Committee of The First Affiliated Hospital of Wenzhou Medical University (approval no. 2012‐57). The *EVA1A* fragments per kilobases million (FPKM) reads expression value was obtained from TCGA data portal to verify the expression difference and explore the clinical significance of *EVA1A* in PTC.

### 2.2 Cell lines and cell culture

TPC‐1 was obtained as a gift from Professor Mingzhao Xing (John Hopkins University School of Medicine). The KTC‐1 and the normal thyroid (HTORI3) cell lines were purchased from the Chinese Academy of Sciences. The cell lines were cultured in RPMI 1640 (Invitrogen; Thermo Fisher Scientific, Inc) and supplemented with 10% foetal bovine serum (FBS, Invitrogen; Thermo Fisher Scientific, Inc). All cells were maintained in humidified and ambient air supplemented with 5% carbon dioxide. The TPC‐1 and the KTC1 cells were seeded into six‐well plates at concentrations of 6 × 10^5^ and 8 × 10^5^ cells/well, respectively, and incubated overnight in the growth medium.

### 2.3 RNA extraction and qRT‐PCR

The Trizol reagent (Invitrogen; Thermo Fisher Scientific, Inc) was used to extract the total RNA. The ReverTra Ace qPCR RT Kit (Toyobo Life Science) was used in accordance with the manufacturer's instructions to reverse transcribe 1 μg of total RNA per sample. The ABI 7500 Fast Real‐Time PCR System (Applied Biosystems; Thermo Fisher Scientific Inc) and the SYBR Green Real‐time PCR Master Mix (TOYOBO, QPK‐201‐201T) were used for qRT‐PCR following the user guide's protocol. The primer sequences used were as follows: *EVA1A* forward primer: 5′‐CGTGGAGATGGCTTTGCTCA‐3′, *EVA1A* reverse primer, 5′‐AGCTGCTCGCTCAGGATTTT‐3′; GADPH forward primer: 5′‐GGTCGGAGTCAACGGATTTG‐3′, GADPH reverse primer: 5′‐ATGAGCCCCAGCCTTCTCCAT‐3′.

### 2.4 RNA interference

The siRNA and the non‐targeting siRNA (negative control [NC]siRNA) used for gene knockdown were obtained from Genepharma (Genepharma). The siRNA sequences of *EVA1A* and negative control siRNA were as follows: *EVA1A* Sense‐1: 5′‐CCUAGCGGCCUAUUCCUUUTT‐3′; *EVA1A* Antisense‐1: 5′‐AAAGGAAUAGGCCGCUAGGTT‐3′; *EVA1A* Sense‐2: 5′‐GAGCCUGAAUCGCUACUAUTT‐3′; and *EVA1A* Antisense‐2: 5′‐AUAGUAGCGAUUCAGGCUCTT‐3′. The cell lines were seeded in six‐well plates for 24 hours, and transfection was performed. The knockdown efficacy was determined using qRT‐PCR.

### 2.5 Cell proliferation assay

Cell proliferation was determined using the Cell Counting Kit‐8 (CCK‐8, Beyotime) in accordance with the manufacturer's protocol. The KTC‐1 and the TPC‐1 cells were seeded into a 96‐well plate (40 196, Cyagen) at a density of 1.5 × 10^3^ cells/well. Each well was added with 10 μL CCK‐8 solution for 3 hours, and the absorbance was measured at 450 nm by using the SpectraMax M5 (Molecular Devices LLC). The same procedure was repeated, and results were measured at 1, 2, 3 and 4 days. All experiments were conducted in triplicate.

### 2.6 Colony formation assay

The two transfected or control cells (1.5 × 10^3^ cells for KTC‐1 and TPC‐1) were seeded into six‐well plates and incubated for 7‐14 days. Each well was fixed with 4% paraformaldehyde (Sigma‐Aldrich Co.) for 30 minutes, stained with 0.01% crystal violet and photographed. All experiments were performed in triplicate.

### 2.7 Cell migration and invasion assays

The migration assay was performed using the Transwell chambers (Corning Incorporated). For the wound healing assay, 1.5 × 10^5^ cells were cultured into a 24‐well plate until 90% confluency was reached. Scratch wounds were created using 0.2 mL pipette tips, and the cells were cultured in medium without serum for 24 hours. Subsequently, the wound healing results were observed under a phase‐contrast inverted cell culture microscope (DMi1, Leica). For the Transwell migration assay, two PTC cells (3.5 × 10^5^ for KTC‐1 and TPC‐1) were suspended in 300 µL serum‐free RPMI1640 and seeded into the upper chamber of each insert. Then, 600 μL RPMI1640 containing 10% FBS was added to the lower chamber of the 24‐well plate. After 24 hours of incubation at 37°C, a cotton swab was used to remove the non‐migrated cells, and the migratory cells on the lower surface were fixed and stained for 30 minutes with 0.01% crystal violet. Invasion assays were performed in the Matrigel Invasion Chambers. For invasion assays, the Matrigel Invasion Chambers (BD Biosciences) were used in accordance with the manufacturer's instructions. The same protocol as the migration assay was done with the invasion chambers.

### 2.8 Apoptosis assay

Cells were collected after 48 hours transfection and stained with 5 μL Annexin V–fluorescein isothiocyanate and 5 μL propidium iodide (BD Biosciences) for 15 minutes. Stained cells were analysed using the FACSCalibur flow cytometer (BD, USA).

### 2.9 GSEA

The GSEA software via the JAVA program (GSEA v.4.0.3, http://www.broadinstitute.org/gsea) was used to analyse the potential genes influenced by the high *EVA1A* expression. The patients with PTC were divided into two groups in accordance with their *EVA1A* expression level (top 50% [high] vs bottom 50% [low]). GSEA was performed to evaluate the effects of *EVA1A* expression levels on various biological gene sets. The gene set groups used in the analysis were obtained from the MSigDB (Molecular Signatures Databases, v7.0, https://www.gsea-msigdb.org/gsea/msigdb/index.jsp).

### 2.10 Protein extraction and Western blot analysis

The transfected cells were lysed in cell lysis buffer (Beyotime) on ice for 30 minutes. Protein concentrations were measured using the bicinchoninic acid assay (Thermo Scientific). The proteins (20 μg) were electrophoresed in 10% SDS–PAGE gels and electrotransferred onto polyvinylidene difluoride membranes (ISEQ00010, Millipore). Afterwards, 5% non‐fat skim milk (BD, Difco™ Skim Milk, 232100) was used to block the membranes in Tris‐buffered saline with Tween (TBST) buffer. The membranes were incubated with the desired primary antibody overnight at 4°C and with the appropriate horseradish peroxidase (HRP)‐conjugated secondary antibodies for 1 hour at room temperature. After each procedure, the membrane was washed thrice with TBST buffer for 10 minutes. The band intensities were quantified using the Image Lab software. The primary antibodies used included vimentin (Abcam), N‐cadherin (Abcam), Bcl‐xL (Abcam), Bax (Proteintech Group), YAP (Proteintech Group), TAZ (Proteintech Group), EVA1A (Bioss) and β‐actin (Proteintech Group). The goat antirabbit HRP‐conjugated IgG (Solarbio) and the goat antimouse HRP‐conjugated IgG (Solarbio) were used as the secondary antibodies. β‐actin served as the internal control.

### 2.11 Statistical analysis

Statistical analysis was accomplished using the SPSS software (SPSS version 22.0, SPSS). To evaluate the diagnostic values of *EVA1A*, SPSS was used to plot the receiver operating characteristic (ROC) curve and calculated the area under the parametric curve (AUC). Graphs were generated using the GraphPad Prism7 (GraphPad Software, Inc, La Jolla, CA, USA), and the normal distribution data were expressed as mean ± standard deviation. Data were analysed using Student's *t* test, paired *t* test and ANOVA (one‐way ANOVA, Student‐Newman‐Keuls). *P*‐values < .05 were regarded as statistically significant.

## 3 RESULTS

### 3.1 *EVA1A* expression is overexpressed in PTC

**Figure 1:**
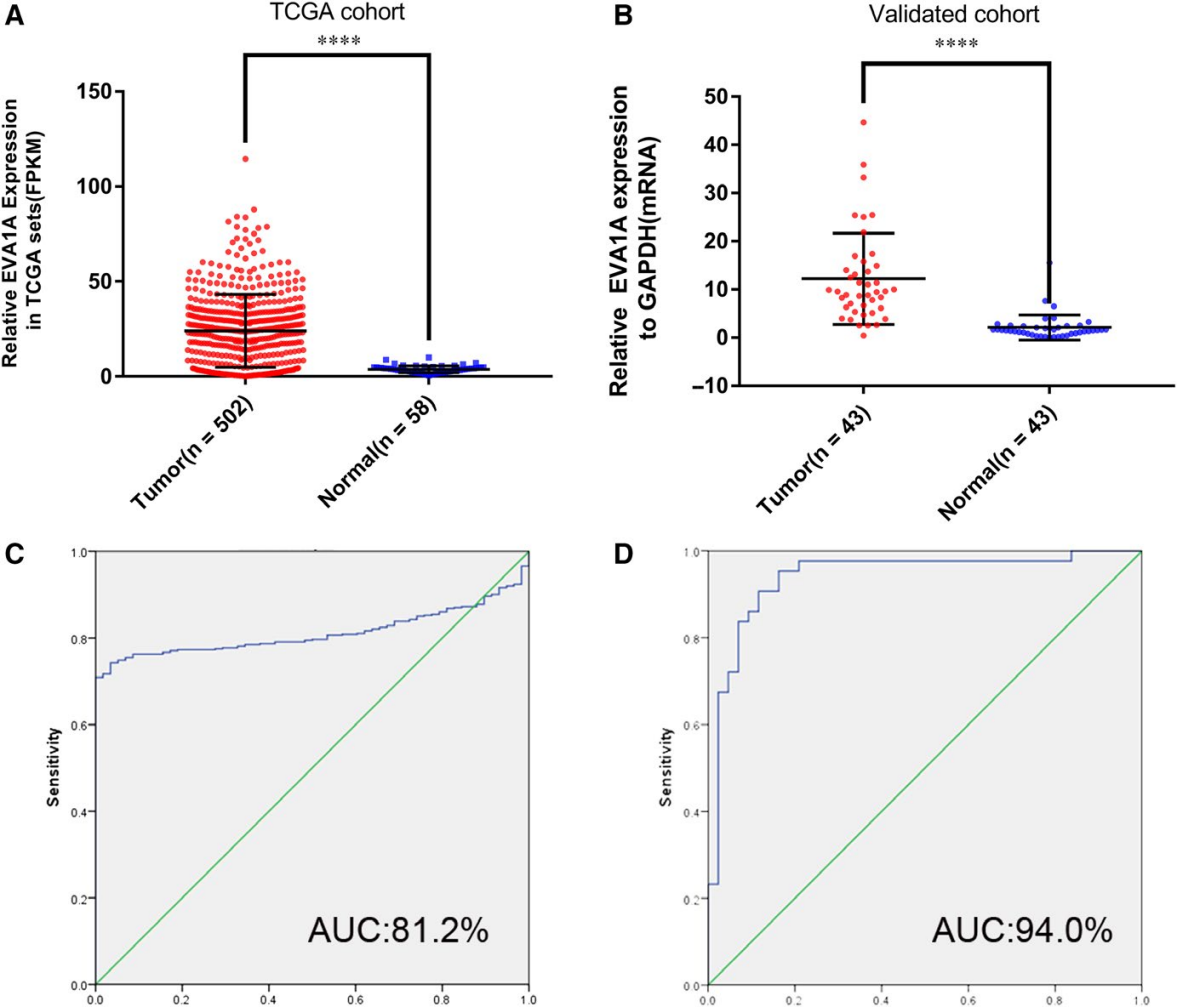
*EVA1A* expression is up‐regulated in PTC samples than in normal thyroid tissues in TCGA and validated cohorts. A, *EVA1A* expression was significantly up‐regulated in the PTC samples than in the TCGA cohort (*P* < .0001). B, *EVA1A* expression was significantly up‐regulated in 43 paired tumours and adjacent tissue as our cohort (*P* < .0001). C, ROC curve for the expression of *EVA1A* in the diagnosis of PTC in the TCGA cohort. AUC: 81.2%, sensitivity: 70.9% and specificity: 100%. D, ROC curve for the expression of *EVA1A* in the diagnosis of PTC in the validated cohort. AUC: 94.0%, sensitivity: 95.3% and specificity: 83.7%. The optimal cut‐off value of the *EVA1A* expression was based on the ROC curve analysis and corresponding Youden index. Data are presented as mean ± standard deviation of three independent experiments. ****P < .0001. *EVA1A*, Eva‐1 homolog A; PTC, papillary thyroid carcinoma; FPKM, Fragments per Kilobase Million; TGCA, The Cancer Genome Atlas; ROC, receiver operating characteristic; and AUC, area under the curve

In our previous study, RNA sequencing analysis is performed on the primary PTC and the adjacent normal tissues obtained from 19 patients with PTC.^11^ After analysing the data, we have found that *EVA1A* was significantly up‐regulated (Table 1). This result coincided with the data shown in the TCGA cohort (Figure 1A). Afterwards, we detected the relative expression of *EVA1A* in 43 patients via qRT‐PCR to verify the sequencing results. The expression level of *EVA1A* in tumour tissues was significantly higher compared with that in adjacent normal tissues (Figure 1B). The ROC curve analysis was used to evaluated the diagnostic capability of *EVA1A*. As shown in Figure 1C,D, *EVA1A* expression had excellent diagnostic value to distinguish non‐cancerous tissue from cancer tissue in the TCGA data set (AUC = 81.2%) and our local cohort (AUC = 94%).

**Table 1:** The expression of EVA1A gene in 19 cases of thyroid papillary carcinoma and paired normal tissue by whole‐transcriptome sequencing

Symbol	RT‐FPKM	RN‐FPKM	Log 2 ratio (RT/RN)	RT/RN
EVA1A	3.639191514	1.398446077	1.37979334993628	Up
EVA1A	4.071582362	2.048957956	0.990699205189628	Up
EVA1A	3.664793903	0.178512116	4.35963816630976	Up
EVA1A	2.750393908	0.662862295	2.05285715764147	Up
EVA1A	4.708554638	1.195500246	1.97766984383549	Up
EVA1A	3.759997596	0.045583129	6.36608796917558	Up
EVA1A	4.16531628	0.273522089	3.92869679470388	Up
EVA1A	3.635602594	0.023623185	7.26584720815565	Up
EVA1A	3.545631674	1.146812799	1.62841276353343	Up
EVA1A	3.060659606	1.330455435	1.20192241657899	Up
EVA1A	3.163299646	1.262942798	1.32464092557588	Up
EVA1A	2.437443349	1.374832405	0.826112926412473	Up
EVA1A	4.894335392	0.679178496	2.8492502813086	Up
EVA1A	3.470146531	1.038905709	1.739931862281	Up
EVA1A	3.253313008	1.532651533	1.08587991375787	Up
EVA1A	4.125607441	0.694351027	2.57086945039085	Up
EVA1A	3.673805665	0.685042923	2.42300902203941	Up
EVA1A	4.63528052	0.39507514	3.55245767808726	Up
EVA1A	3.93988209	0.806681259	2.28808180995356	Up

Abbreviations: FPKM, Fragments Per Kilobase Million; RN, RNA normal tissues; RT, RNA tumour tissues.

### 3.2 *EVA1A* is associated with the clinicopathological features of PTC

TCGA data were analysed to determine the relationship between the expression level of *EVA1A* and the clinicopathological features and further explore the role of *EVA1A* in PTC. We divided the patients with PTC into two groups, namely high (n = 251) and low (n = 251) *EVA1A* expression in accordance with the median value of the *EVA1A* expression level in TCGA cohort. Results showed that a high *EVA1A* expression was related to histological type (*P* < .001), age (*P* = .025), tumour size (*P* = .06) and lymph node metastasis (LNM; *P* < .001; Table 2). Nevertheless, the associations of *EVA1A* expression with gender, distant metastasis and disease stage (AJCC7) were not significant. In our validated cohort, high *EVA1A* expression was correlated with LNM (*P* = .009) and disease stage (AJCC7; *P* = .011; Table 3). These results supported that *EVA1A* gene was an oncogene in PTC.

**Table 2:** The association between EVA1A expression and clinicopathologic features in the TCGA cohort

Clinicopathologicfeatures	High expression (n = 251)	Low expression (n = 251)	Χ^2^	*P*‐value
Gender			0.496	.481
Female	64	71		
Male	187	180		
Age (y)			5.036	.025*
Median (mean ± SD)	45.46 ± 15.82	49.22 ± 15.65		
<60	205	184		
≥60	46	67		
Histological type			30.288	<.001*
Classical	206	150		
Other types	45	101		
Tumour size (mm)			3.53	.06
≥20	189	170		
<20	62	81		
Lymph node metastasis			27.167	<.001*
Yes	140	82		
No	111	169		
Distant metastasis				.504
Yes	6	3		
No	245	248		
Disease stage (AJCC7)			2.019	.155
I + II	160	175		
III + IV	91	76		

Abbreviations: AJCC7, Seven Edition of American Joint Committee on Cancer; TCGA, The Cancer Genome Atlas.

* Chi‐square test, *P*‐value < .05.

**Table 3:** The association between EVA1A expression and clinicopathologic features in the local cohort

Clinicopathologicfeatures	High expression (n = 21)	Low expression (n = 22)	*P*‐value
Tumour size (mm)			.203
≥20	5	10	
<20	16	12	
Lymph‐node metastasis			.014*
Yes	16	8	
No	5	14	
Disease stage (AJCC7)			.016*
I + II	12	20	
III + IV	9	2	

Abbreviations: AJCC7, Seven Edition of American Joint Committee on Cancer.

* Chi‐square test, *P*‐value < .05.

### 3.3 *EVA1A* overexpression aggravates the risk of LNM in PTC

Further investigation was carried out to find the relationship of *EVA1A* expression with LNM. We conducted a univariate logistic regression analysis in TCGA data. Results showed that the significant variables for LNM were *EVA1A* expression (odds ratio [OR] = 2.599, 95% confidence interval [CI] = 1.808‐3.736, *P* < .001), histological type (OR = 0.378, 95% CI = 0.249‐0.573, *P* < .001), gender (OR = 0.63, 95% CI = 0.424‐0.937, *P* = .023), disease stage (AJCC7; OR = 3.692, 95% CI = 2.498‐5.458, *P* < .001) and tumour size (OR = 2.313, 95% CI = 1.530‐3.495, *P* < .001; Table 4). The multivariate logistic analysis validated that *EVA1A* expression (OR = 2.199, 95% CI = 1.475‐3.278, *P* < .001), histological type (OR = 0.354, 95% CI = 0.221‐0.569, *P* < .001), disease stage (AJCC7; OR = 3.441, 95% CI = 2.240‐5.284, *P* < .001), tumour size (OR = 1.748, 95% CI = 1.099‐2.781, *P* = .018) and multi‐nodularity (OR = 1.535, 95% CI = 1.028‐2.292, *P* = .036) were significant and independent high‐risk factors of LNM (Table 5). In brief, the high expression level of *EVA1A* can indicate the independent high risk of LNM in PTC.

**Table 4:** Univariate logistic regression analysis for the risk of lymph node metastasis

Factor	OR	95% CI	*P*‐value
EVA1A expression (high vs low)	2.599	1.808‐3.736	<.001*
Histological type	0.378	0.249‐0.573	<.001*
Age, y (≤60 vs >60)	0.654	0.425‐1.008	.054
Gender (male vs female)	0.63	0.424‐0.937	.023*
Disease stage (AJCC7)	3.692	2.498‐5.458	<.001*
Tumour size (mm)	2.313	1.530‐3.495	<.001*
Multi‐nodularity	1.435	1.006‐2.046	.046*

Abbreviations: AJCC7, Seven Edition of American Joint Committee on Cancer.

* Chi‐square test, *P*‐value < .05.

**Table 5:** Multivariate logistic regression analysis for the risk of lymph node metastasis

Factor	OR	95% CI	*P*‐value
EVA1A expression (high vs low)	2.199	1.475‐3.278	<.001*
Histological type	0.354	0.221‐0.569	<.001*
Gender (male vs female)	0.735	0.471‐1.147	.175
Disease stage	3.441	2.240‐5.284	<.001*
Tumour size (mm)	1.748	1.099‐2.781	.018*
Multi‐nodularity	1.535	1.028‐2.292	.036*

Abbreviations: AJCC7, Seven Edition of American Joint Committee on Cancer.

* Chi‐square test, *P*‐value < .05.

### 3.4 *EVA1A* knockdown suppresses cell proliferation and colony formation

**Figure 2:**
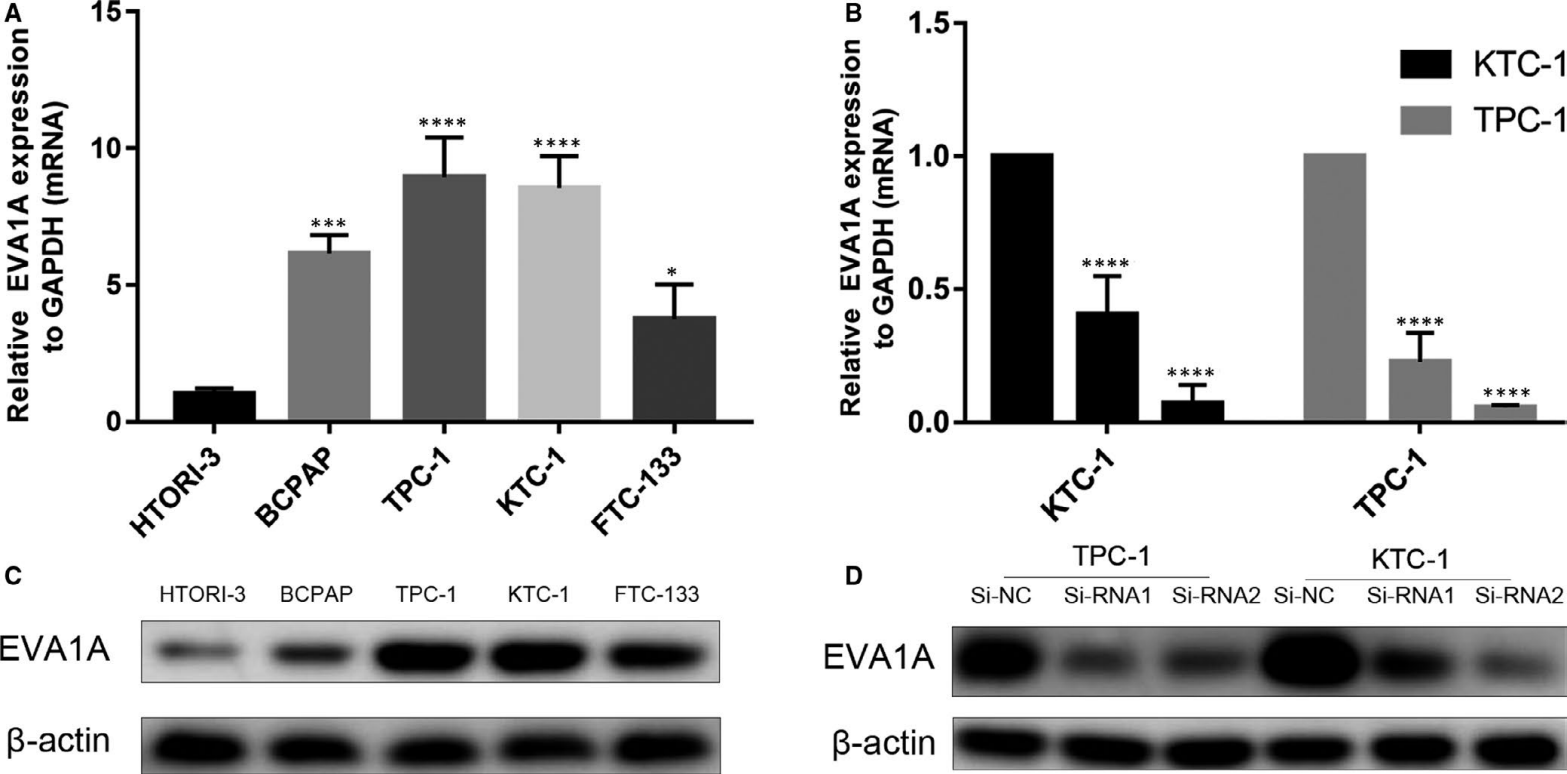
*EVA1A* expression in PTC cell lines and Si‐RNA transfection efficiency in TPC‐1 and KTC‐1. A, Relative expression of *EVA1A* (compared with GAPDH) in the normal thyroid cell line HTORI‐3 and three PTC cells. B, *EVA1A* relative expression (compared with GAPDH) was detected in the TPC‐1 and the KTC‐1 cell lines transfected with siRNA by qRT‐PCR. C, Relative protein expression of *EVA1A* determined using Western blot analysis in the normal thyroid cell line HTORI‐3 and four PTC cell lines. D, *EVA1A* relative protein expression was detected using Western blot analysis in transfected TPC‐1 and KTC‐1 cells. Data are presented as mean ± standard deviation of three independent experiments. *P < .05; ***P < .001; ****P < .0001 in comparison with the control group using Student's *t* test. *EVA1A*, Eva‐1 homolog A; PTC, papillary thyroid cancer; RNA‐seq, RNA sequencing; and RT‐qPCR, real‐time quantitative polymerase chain reaction

**Figure 3:**
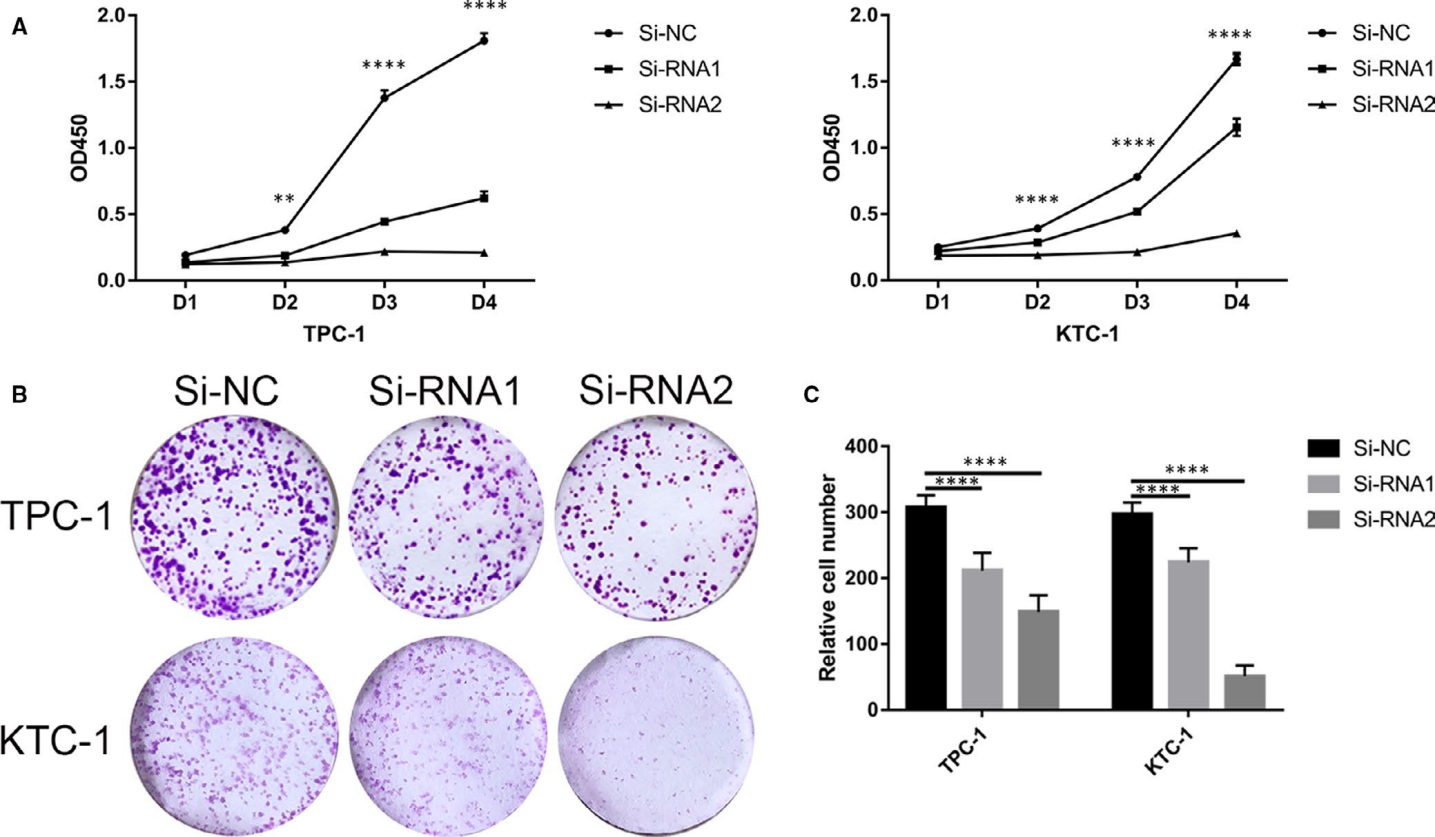
Knockdown of *EVA1A* inhibits PTC cell line proliferation. A, Cell proliferation assay. 1.5 × 10^3^ TPC1 and KTC‐1 cells transfected with siRNA or Si‐NC were cultured into 96‐well plates for 1‐4 days, and cell proliferation was measured using the Cell Counting Kit‐8 (CCK‐8) assay. B, Colony formation assay. The transfected cells were seeded into six‐well plates, incubated for more than 7 days and stained with 0.01% crystal violet. Compared with Si‐NC proliferation, Si‐RNA1 and Si‐RNA2 proliferation were significantly suppressed in PTC cells. C, Relative quantification of the colony numbers in the (B) colony formation assay. Data are presented as mean ± standard deviation of three independent experiments. **P < .01, ****P < .0001 in comparison with the control group using Student's *t* test

We evaluated the expression level of *EVA1A* in several PTC and normal thyroid cell lines (HTORI‐3) in the mRNA and protein levels. Results showed that *EVA1A* expression was higher in TPC‐1, KTC‐1, BCPAP and FTC‐133 than that in HTORI‐3 (Figure 2A,C). TPC‐1 and KTC‐1 were used to determine the function of *EVA1A* in PTC because of their high *EVA1A* expression. The *EVA1A* expression was down‐regulated by siRNA‐1 and siRNA‐2. The mRNA and protein level expression of *EVA1A* were significantly down‐regulated after transfection (Figure 2B,D). Thereafter, we performed the CCK‐8 and the colony formation assays in TPC‐1 and KTC‐1. The down‐regulation of *EVA1A* significantly inhibited the cell proliferation in both assays (Figure 3).

### 3.5 *EVA1A* knockdown inhibits migration and invasion

**Figure 4:**
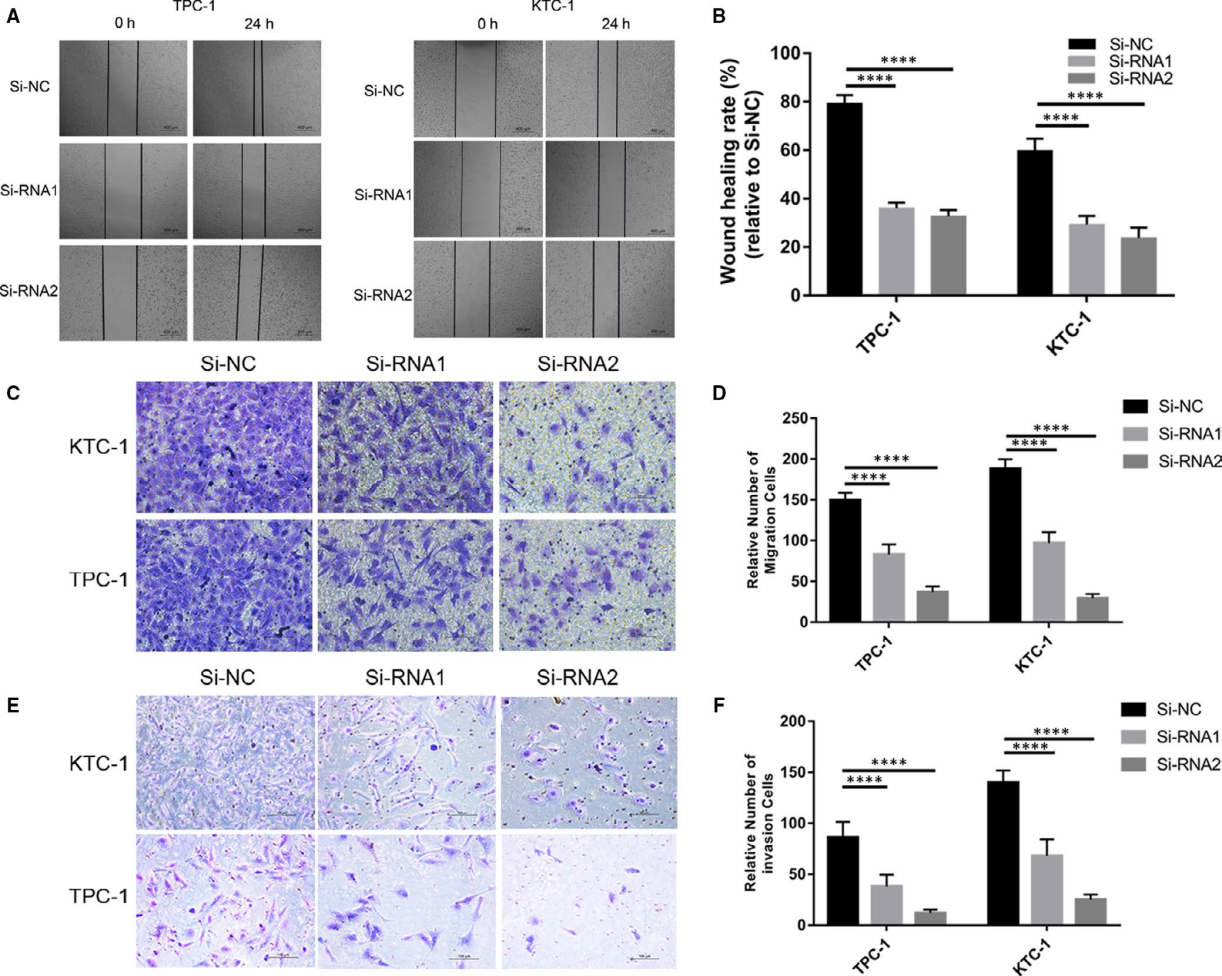
*EVA1A* down‐regulation inhibits cell migration and invasion in PTC cells. A, Cell migration was determined in PTC cells transfected with Si‐RNA through the wound healing assay. Images were captured at 0 and 24 h. B, Quantification of the wound healing assay. The column shows the 24 h intercellular distance normalized with 0 h as 100%. Wound healing rate = (0 h wound area − 24 h wound area)/0 h wound area × 100%. C, E, Transwell migration and invasion assays. The migration and invasion of PTC cells were suppressed by the down‐regulation of *EVA1A* (compared with Si‐NC). D, F Quantitative results of cell numbers. The data shown in the graph are the cell numbers from three independent experiments. ****P < .0001 in comparison with the control group using Student's *t* test

The migration and invasion assays were performed on TPC‐1 and KTC‐1 to further confirm the function of *EVA1A* in PTC. As expected, the PTC cells with underexpressed *EVA1A* had significantly inhibited ability to migrate (Figure 4A,C) and invade (Figure 4E) compared with those in the Si‐NC control group.

### 3.6 *EVA1A* underexpression effectively induces PTC cell line apoptosis in vitro

**Figure 5:**
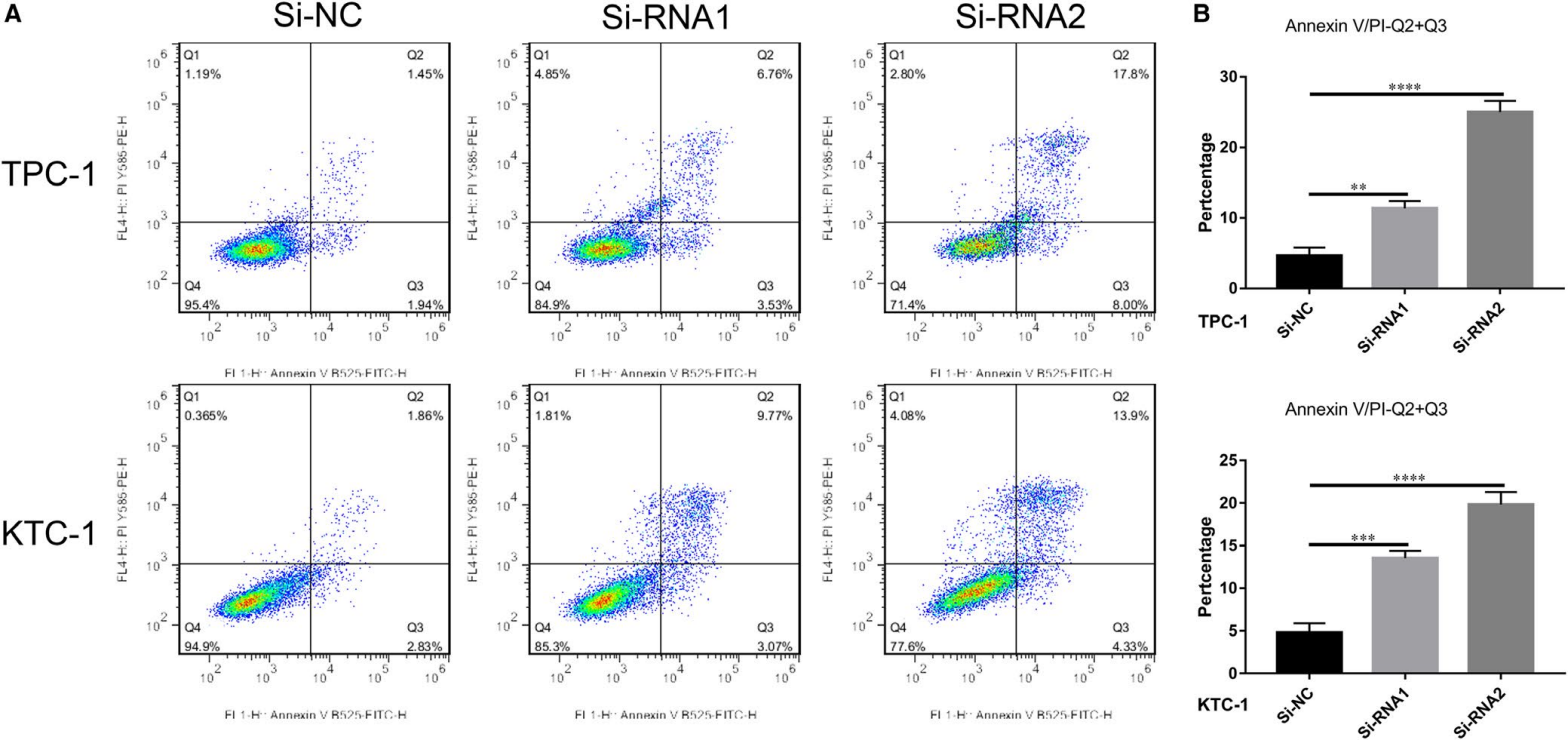
*EVA1A* gene knockdown induces PTC cell apoptosis in vitro. A, Flow cytometry analysis unveiled the apoptosis rates of the TPC‐1 and the KTC‐1 cells transfected with Si‐NC and Si‐RNA. B, Quantitative results of the apoptosis cell percentages. Cell apoptosis includes early (Q3) and (Q2) late apoptosis. Percentage of cell apoptosis = Q2 + Q3. **P < .01, ***P < .001, ****P < .0001, Data are presented as mean ± standard deviation

Flow cytometry was used to detect the proportion of apoptotic transfected cell lines to explore whether *EVA1A* plays a role in cell cycle. Results indicated that the cell lines with *EVA1A* knockdown enhanced PTC cell (TPC‐1 and KTC‐1) apoptosis compared with the Si‐NC cell lines (Figure 5).

### 3.7 *EVA1A* knockdown induces the activation of the intrinsic apoptosis pathway and suppresses EMT progression via the Hippo signalling pathway

**Figure 6:**
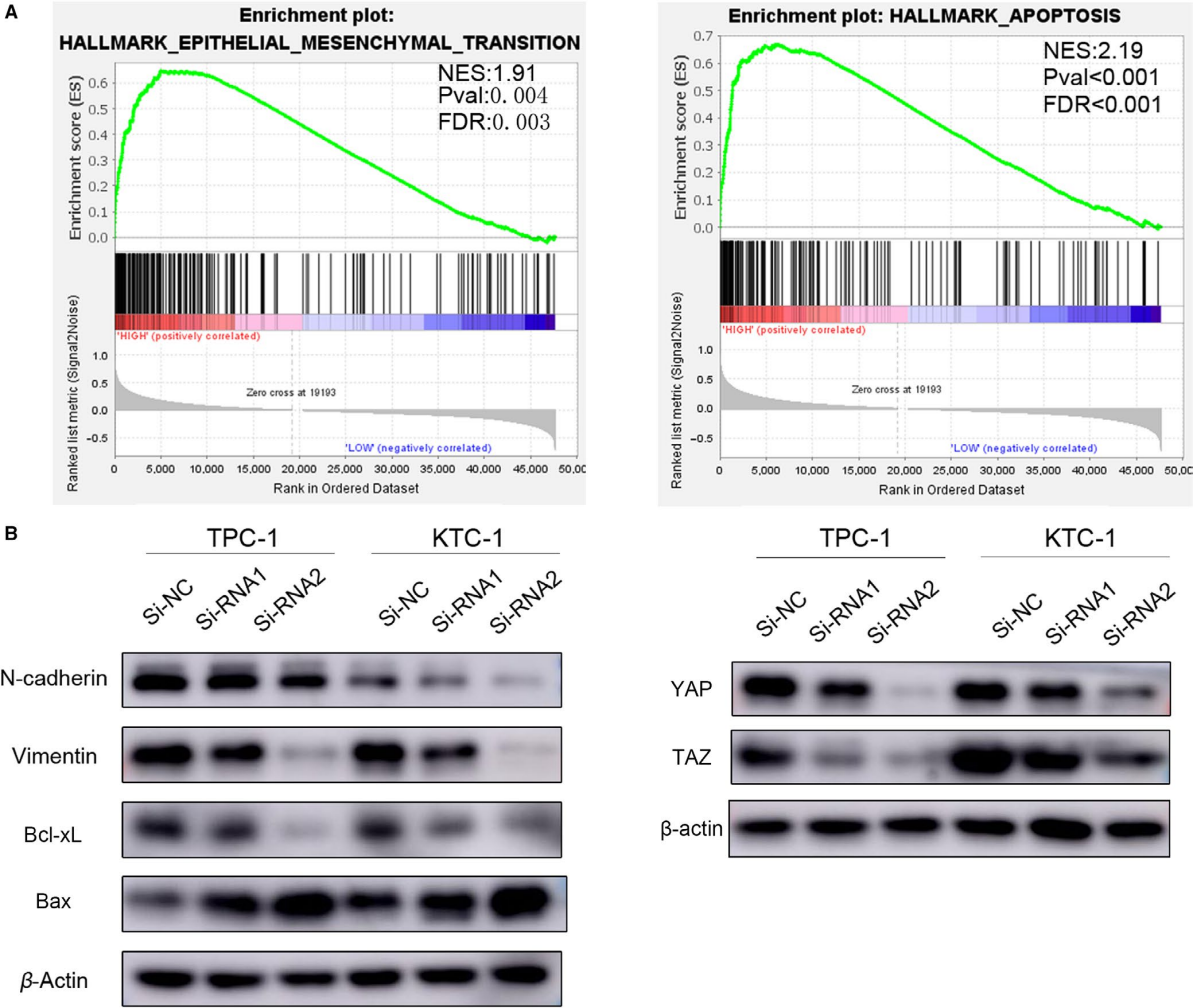
*EVA1A* knockdown induces the activation of the intrinsic apoptosis pathway and suppresses EMT progression via the Hippo signalling pathway. A, Gene set enrichment analysis (GSEA) plots showing that the high expression of *EVA1A* was positively correlated with the epithelial‐mesenchymal transition (EMT) signalling pathway (NES:1.91, Pval:0.004, FDR:0.003) and apoptosis (NES:2.19, *P* < .001, FDR < 0.001). B, Levels of N‐cadherin, vimentin, Bcl‐xL, Bax, YAP and TAZ were evaluated using Western blot with β‐actin serving as a loading control

Accumulating number of studies have shown that EMT and apoptosis plays a critical role in PTC metastasis and proliferation.^25^, ^26^ GSEA indicated that the expression of *EVA1A* was related to EMT and apoptosis (Figure 6A). Related proteins were detected to explore the mechanism of the *EVA1A* on EMT and apoptosis progression. Recent studies have provided enormous evidences suggesting that the Hippo signalling pathway can induce EMT.^17^, ^27^, ^28^ Results revealed that compared with the control group, the Si‐*EVA1A* group showed decreased N‐cadherin, vimentin and Bcl‐xL expression and increased Bax expression (Figure 6B). Furthermore, the YAP and TAZ levels in the Si–*EVA1A* group were lower than those in the control group (Figure 6B). These results suggested that *EVA1A* may inhibit apoptosis and promote the metastasis and the proliferation of PTC by suppressing the intrinsic apoptosis pathway and activating the EMT via the Hippo signalling pathway.

## 4 DISCUSSION

With the development of molecular biology, our understanding of PTC molecular mechanisms is gradually developing. Next‐generation sequencing has paved the way for the discovery that PTC is driven by several characteristic genetic alterations, such as point mutations in proto‐oncogenes and chromosomal rearrangements.^29^ However, despite the enormous progress in PTC genetic research, the current non‐surgical treatment for thyroid cancer is still limited. Therefore, further research on the pathogenetic mechanism of PTC is essential for the diagnosis and therapy of PTC.

According to Cancer Statistics 2019, thyroid cancer incidence is rising faster than other cancers in men and women and accounts for 4% of the total estimated female cancer burden in 2019.^30^ In our previous study, whole‐genome sequencing has been performed on 19 pairs of PTC tumours and adjacent normal tissues.^11^ We have found that the expression level of *EVA1A* is up‐regulated in patients with PTC in Wenzhou. This finding supports the hypothesis that *EVA1A* may have a role as an oncogene in PTC. The function of *EVA1A* in apoptosis is complex and suggested as essential in the process. However, the molecular mechanisms of *EVA1A* in thyroid carcinoma are still unclear.

In the present study, the statistical results of the clinicopathologic feature analysis in TCGA and the local cohorts of PTC showed that the up‐regulation of *EVA1A* was related to LNM. The results of the in vitro assays also verified the assumption that *EVA1A* was a novel oncogene in PTC. The down‐regulation of *EVA1A* can significantly inhibit PTC cell proliferation and colony formation. Also, *EVA1A* down‐regulation can weaken cell migration and invasion in PTC cell lines. Apoptosis was evaluated via flow cytometry, and results showed that the down‐regulation of *EVA1A* can enhance the apoptosis of PTC cells. According to the GSEA analysis, the apoptosis and EMT signalling pathways were significantly correlated with high expression of *EVA1A*. The results of Western blotting showed that the underexpression of *EVA1A* inhibit the protein level of vimentin, Bcl‐xL, YAP, and TAZ and enhance Bax. This finding suggested that *EVA1A* can promote the metastasis and proliferation and inhibit the apoptosis of PTC by regulating the intrinsic apoptosis pathway and EMT via the Hippo signalling pathway.

In summary, we first found that *EVA1A* in PTC was a potent oncogene of enhanced tumour aggressiveness. This evidence suggested that *EVA1A* may be an effective molecular marker of PTC for diagnosis and therapy.

## CONFLICT OF INTEREST

The authors declare that they have no conflict of interest.

## AUTHOR CONTRIBUTION

**Bangyi Lin:** Methodology (lead); Writing‐original draft (lead). **Jia‐Liang Wen:** Data curation (lead); Formal analysis (lead). **Chen Zheng:** Writing‐review & editing (lead). **Lizhi Lin:** Data curation (supporting); Formal analysis (supporting); Methodology (supporting). **Cheng‐Ze Chen:** Writing‐review & editing (supporting). **Jin‐Miao Qu:** Conceptualization (lead); Funding acquisition (lead); Project administration (lead); Writing‐review & editing (supporting).

## ETHICAL APPROVAL

The ethical approval for this study was obtained from the Ethics Committee of the First Affiliated Hospital of Wenzhou Medical University.

## INFORMED CONSENT

Written informed consent was obtained from each participant (approval no. 2012‐57).

## Data Availability

The data sets used during this study and additional images are included in this article. Raw data are available upon reasonable request.
